# Routine Inflammation–Haemostasis Panel in Transvenous Lead Extraction: An Exploratory Prospective Single-Centre Cohort Study

**DOI:** 10.3390/jcm15176923

**Published:** 2026-09-07

**Authors:** Jakub K. Rokicki, Sylwia Wolff, Łukasz Januszkiewicz, Marcin Michalak, Katarzyna Czarzasta, Andrzej Cacko, Grzegorz Opolski, Marcin Grabowski

**Affiliations:** 1 Department of Medical Informatics and Telemedicine, Medical University of Warsaw, 00-587 Warsaw, Poland; 2 First Department of Cardiology, Medical University of Warsaw, 00-587 Warsaw, Poland; 3 National Oncology Institute of Maria Sklodowska-Curie—National Research Institute in Warsaw, 01-045 Warsaw, Poland; 4 Department of Physiology and Pathophysiology, Laboratory of Centre for Preclinical Research, Medical University of Warsaw, 00-587 Warsaw, Poland

**Keywords:** transvenous lead extraction, coagulation, inflammation, haemostasis, risk stratification, cardiac implantable electronic device infection, matrix metalloproteinase-9, decision-curve analysis

## Abstract

**Background/Objectives:** Vascular and bleeding complications drive procedural mortality in transvenous lead extraction (TLE), yet contemporary risk scores include no pre-procedural laboratory measure of haemostasis or inflammation. We evaluated whether a routine inflammation–haemostasis panel (coagulation tests, blood counts and C-reactive protein [CRP]) and peri-procedural matrix metalloproteinase-9 (MMP-9) dynamics predict technical intra-procedural complexity. **Methods:** Fifty-four procedures in 53 consecutive patients at a single tertiary centre were analysed prospectively (50% infective; 86 leads). Technical complexity (prolonged procedure or fluoroscopy time; advanced-tool use) was modelled with the number of extracted leads as a predictor rather than an outcome component, in prespecified and stepwise-selected logistic models with bootstrap optimism correction. **Results:** The infective indication predicted extraction extent (≥2 leads: 70% vs. 22%) but not technical complexity: the time-based endpoint was not associated with the indication (OR 0.38, *p* = 0.16), and only cumulative lead dwell time predicted advanced-tool use (OR 1.27 per year; optimism-corrected area under the curve [AUC] 0.72). No routine panel variable predicted any technical endpoint, and none survived multiplicity correction. MMP-9 neither changed peri-procedurally nor differed between indications. A principal-component composite did not improve on CRP alone (AUC 0.66 vs. 0.69; *p* = 0.67), and the full panel’s apparent AUC (0.84) fell to 0.71 after optimism correction. **Conclusions:** No routine pre-procedural inflammation–haemostasis variable predicted technical complexity; the infective indication’s apparent association reflected guideline-mandated complete system removal—extraction extent—rather than technical difficulty. These exploratory findings support dwell-time- and anatomy-anchored risk scoring, directed haemostasis-activation markers rather than routine screening, and prospective studies capturing bleeding endpoints.

## 1. Introduction

Transvenous lead extraction (TLE) carries a low but non-trivial risk of major vascular and bleeding complications. In the ESC–EHRA ELECTRa registry, major intra-procedural complications occurred in 1.7% of 3510 procedures, and 49 of 58 major events (84%) required pericardiocentesis, chest-tube placement or surgical repair for cardiovascular injury [1,2]. The thirty-day mortality of TLE performed for systemic infection reaches 4%, and predictors of major complications are dominated by indication (infection), lead dwell time and operator or centre volume [2,3].

Pre-procedural risk stratification for TLE relies on clinical and anatomical scores: the SAFeTY-TLE score (sum of lead dwell times, anaemia, female sex, treatment history, younger age) [4], the Lead Extraction Difficulty (LED) index [5,6], the ELECTRa Registry Outcome Score (EROS) [7], the PADIT infection-risk score [8,9], and the more recent LECOM and machine-learning-derived ELECTRa scores [10,11]. Pre-procedural multidetector computed tomography is increasingly used to refine anatomical risk [12,13]. None of these tools includes a circulating laboratory marker of haemostasis or inflammation, although vascular bleeding dominates the mortality profile of TLE. The clinical impact of peri-procedural antithrombotic therapy was assessed in the ELECTRa sub-analysis by Di Cori and colleagues, which observed a higher rate of minor bleeding events with continued anticoagulation and otherwise similar major-complication rates with minimised antithrombotic therapy [14].

In this prospective single-centre cohort we addressed, in a planned exploratory analysis, the value of these routinely available markers for procedural risk assessment in TLE. Our primary question was whether the routine inflammation–haemostasis panel, with or without matrix metalloproteinase-9 (MMP-9), predicts technical intra-procedural complexity—defined by procedure time, fluoroscopy time and requirement for advanced tools—independently of the infective indication; because complete system removal is mandated in infection, the number of extracted leads is treated as a measure of extraction extent, not of technical difficulty. We secondly characterised peri-procedural MMP-9 dynamics and their relationship to baseline haemostasis and inflammation. Finally, because the infective versus non-infective indication is known clinically before extraction and is therefore not a diagnostic target, we report the between-indication comparison of the panel and of MMP-9—together with a principal-component composite benchmarked against C-reactive protein (CRP) alone—as a descriptive basic-science observation of the peri-procedural inflammatory–haemostatic milieu.

In this prospective single-centre cohort we therefore tested three questions in a planned exploratory analysis. First, does the routine pre-procedural inflammation–haemostasis panel discriminate the infective from the non-infective TLE indication after multiple-testing correction? Second, does a principal-component composite score derived from this panel match or outperform CRP alone? Third, does the panel, with or without MMP-9, predict technical intra-procedural complexity (prolonged procedure or fluoroscopy time; requirement for advanced tools) independently of the infective indication, with the number of extracted leads treated as a predictor of extraction extent?

## 2. Materials and Methods

### 2.1. Study Design and Population

**Figure 1:**
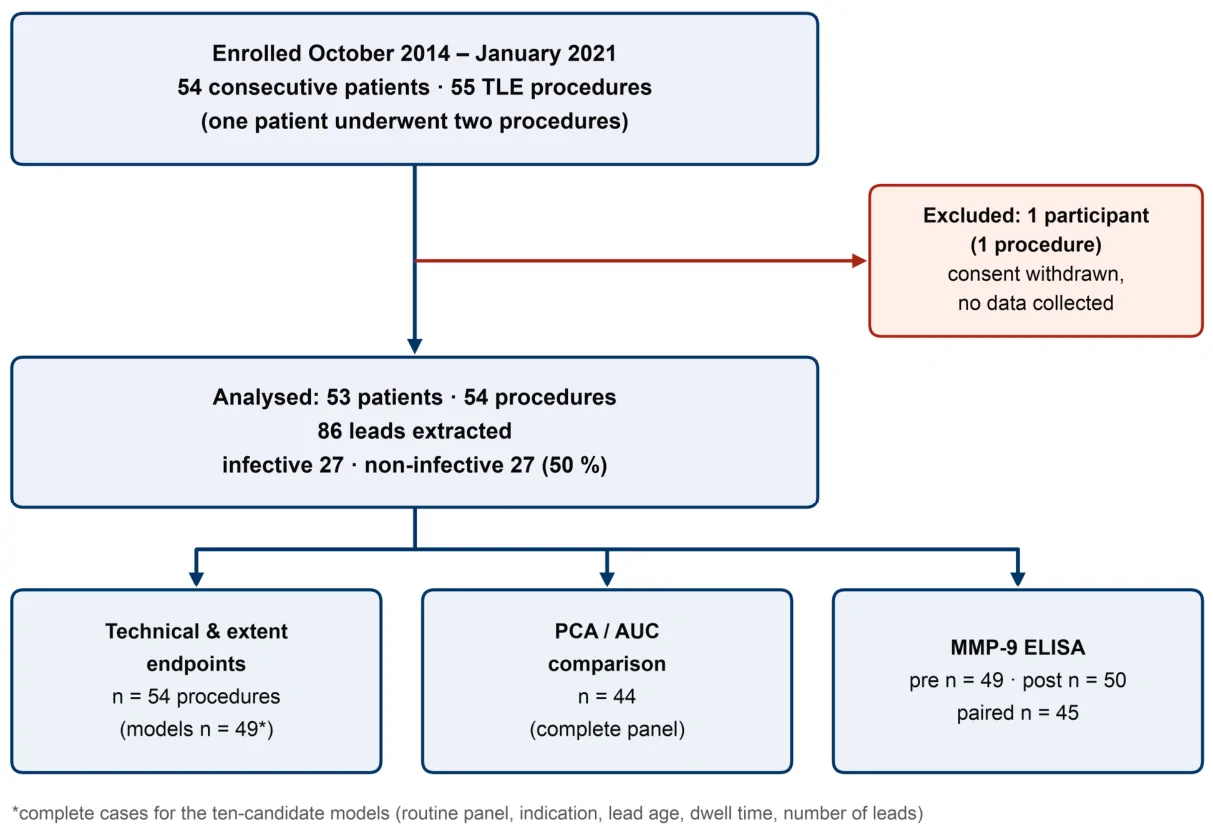
Participant flow. Fifty-four consecutive patients underwent 55 transvenous lead extraction procedures; one participant withdrew consent before completion of data collection and was excluded from all analyses; 54 procedures in 53 patients were analysed. Analysis subsets reflect complete-case availability.

Fifty-four consecutive adults undergoing TLE at the First Department of Cardiology of the Medical University of Warsaw between October 2014 and January 2021 were enrolled prospectively and underwent 55 extraction procedures (one patient underwent two procedures four months apart). One participant withdrew consent before completion of data collection and was excluded from all analyses; 54 procedures in 53 patients were analysed (Figure 1). All patients had a Class I or IIa indication according to the 2018 EHRA expert consensus statement on lead extraction [15] and provided written informed consent. Exclusion criteria were age below 18 years, life expectancy below six months, active malignancy and pregnancy. The study complied with the Declaration of Helsinki and was approved by the Bioethics Committee of the Medical University of Warsaw.

### 2.2. Indication Classification and Procedure

Indications were dichotomised as infective (isolated pocket infection, pocket-related infective endocarditis or lead-dependent infective endocarditis [LDIE]) or non-infective (lead malfunction, lead recall, system upgrade, lead perforation or venous occlusion) according to the 2020 EHRA–ISCVID–ESCMID consensus document [3]. Procedures were performed under general anaesthesia in a hybrid theatre with surgical backup, using a stepwise strategy (simple traction; locking stylets; mechanical dilator sheaths; rotational mechanical sheaths) and were recorded per 2018 EHRA endpoints [15]; procedural outcome was graded accordingly as complete procedural success (radiological and clinical success), clinical success (retained lead fragment not compromising the goals of the procedure) or failure.

### 2.3. Laboratory Measurements

Pre-procedural blood was sampled on the day before TLE; post-procedural blood was sampled on the first morning after extraction. The routine inflammation–haemostasis panel—prothrombin time (PT), international normalised ratio (INR), activated partial thromboplastin time (APTT), platelet count, haemoglobin, total white-cell count and CRP—was measured by the hospital central laboratory using local reference methods. Blood was allowed to clot at room temperature and centrifuged; serum aliquots were stored at −80 °C until assay, and each sample underwent a single freeze–thaw cycle. MMP-9 was quantified in serum by commercial sandwich ELISA (Quantikine^®^ Human MMP-9 immunoassay, catalogue no. DMP900; R&D Systems, Minneapolis, MN, USA) in duplicate, with inter-assay coefficient of variation below 10%. N-terminal pro-B-type natriuretic peptide (NT-proBNP) was measured by the routine laboratory method. SAFeTY-TLE and PADIT scores were calculated for every patient from the clinical record.

### 2.4. Endpoints

Prespecified endpoints were (i) the infective indication for TLE (descriptive between-group comparison only); (ii) technical procedural complexity, definition A—fluoroscopy time above its 75th percentile OR procedure time above its 75th percentile; and (iii) technical procedural complexity, definition B—requirement for the Byrd dilator sheath OR the Evolution rotational dilator sheath. The number of extracted leads was treated as a predictor—a determinant of extraction extent—rather than as an outcome component, because complete system removal is mandated by the infective indication, and its inclusion in a complexity endpoint would incorporate the indication into the outcome. Composite definitions A and B—identical to the technical definitions except that any procedure with ≥2 extracted leads was additionally classified as complex—were analysed as extraction-extent sensitivity endpoints. The exploratory ‘difficult extraction’ proxy is reported only in the Supplementary Materials (Sections S5 and S8) as hypothesis-generating.

### 2.5. Statistical Analysis

Continuous variables are reported as median (interquartile range, IQR); categorical variables as n (%). Between-group comparisons used the Mann–Whitney U test or Fisher’s exact test as appropriate. Standardised mean differences (Hedges’ g) with bias-corrected and accelerated (BCa) bootstrap 95% CIs (2000 resamples), Cliff’s delta with BCa 95% CIs, and Hodges–Lehmann (HL) median differences with 95% CI were used as effect-size measures; rank-based measures are emphasised for skewed variables. Multiplicity was controlled by both the Bonferroni procedure and the Benjamini–Hochberg (BH) procedure across the 10 panel variables. A principal-component analysis (PCA) on z-scored panel variables, with haemoglobin inverted to capture anaemia direction, was used to derive a composite inflammation–haemostasis (IH) score (PC1). PC1 was compared with CRP alone and with the full multivariable panel by logistic regression for the infective indication, with the area under the receiver-operating-characteristic curve (AUC) computed with DeLong 95% CI and pairwise comparison by the DeLong test. Wilcoxon signed-rank tests compared MMP-9 pre- and post-extraction overall and within indication strata. Spearman correlations of MMP-9 percentage change with baseline laboratory values are reported with BH-adjusted q-values. A multivariable linear regression of MMP-9 percentage change on CRP, platelets, haemoglobin, the infective indication, log procedure time and number of leads used HC3 robust standard errors; residual normality was assessed by Shapiro–Wilk. Logistic regression for the procedural endpoints used Bayesian-information-criterion (BIC)-based stepwise selection from CRP, platelets, haemoglobin, PT, APTT, INR, the infective indication, lead age, cumulative lead dwell time and—for the technical endpoints—the number of extracted leads as a predictor; prespecified two-predictor models (the infective indication and cumulative lead dwell time) were additionally fitted for both technical endpoints, with Firth penalised likelihood prespecified in case of separation. Model performance is reported as apparent AUC with DeLong CI and as optimism-corrected AUC after 1000 bootstrap resamples; calibration of the extraction-extent models was assessed by the Hosmer–Lemeshow test and by decision-curve analysis over threshold probabilities 0.20–0.70, and calibration of the technical-endpoint models by the bootstrap-corrected calibration slope. Analyses were performed in MedCalc 16.8.4 (MedCalc Software, Ostend, Belgium) and in Python 3.13.14 (NumPy 2.3.5, pandas 2.2.3, SciPy 1.17.1, statsmodels 0.14.6, scikit-learn 1.6.1) via Julius.ai (Julius Technologies, Middletown, DE, USA). Two-sided *p* < 0.05 was considered statistically significant; given the small sample size, all multivariable analyses are positioned as exploratory. One enrolled participant withdrew consent before completion of data collection and was excluded from all analyses; a sensitivity analysis excluding the second procedure of the one patient who underwent two extractions was performed for the technical-endpoint models. The endpoint decomposition, the technical-endpoint models, bootstrap optimism corrections, BCa effect-size intervals and the sensitivity analyses were performed in Python 3.11.15 (NumPy 2.4.4, pandas 3.0.2, SciPy 1.17.1, statsmodels 0.15.0, scikit-learn 1.8.0) with a fixed random seed.

Sensitivity analyses comprised (i) lasso-penalised logistic regression (α = 1.0) with 5-fold cross-validation as a robustness check on the BIC-based stepwise selection (extraction-extent composite definition A: out-of-fold AUC = 0.767, retained predictors infective indication [standardised β = +0.74], cumulative lead dwell time [+0.41], APTT [+0.34], PT [+0.30] and platelets [+0.10]; composite definition B: out-of-fold AUC = 0.752 with infective indication, cumulative lead dwell time, APTT and platelets retained); (ii) calibration-in-the-large and calibration-slope decomposition with 1000-bootstrap CIs; and (iii) a post hoc Hanley–McNeil power analysis, which estimated the minimum detectable AUC at α = 0.05 and 80% power as 0.681 for the infective endpoint, 0.684 for composite definition A and 0.688 for composite definition B. The routine-panel AUCs of approximately 0.66 reported below sit immediately below this detection floor; the null finding is therefore consistent with limited statistical power rather than with a complete absence of discriminatory information and constitutes a quantitative pre-specification of the sample size required for any prospective replication.

## 3. Results

### 3.1. Cohort

Fifty-three patients underwent 54 extraction procedures (86 leads; Figure 1). Median age was 70 (IQR 60.5–80) years, 67% were male, median cumulative lead dwell time was 5.2 (IQR 2.7–8.2) years, median LVEF was 45.5% (IQR 30–55) and CKD-EPI eGFR < 60 was observed in 58%. Atrial fibrillation was present in 61%, hypertension in 65% and diabetes in 30%. The indication was infective in 27 procedures (50%). The infective indication comprised LDIE in 14 procedures, isolated pocket infection in 12 and bacteraemia without an identified source in one. Procedure time was shorter in the infective group (median 70 vs. 85 min; *p* = 0.036), as was fluoroscopy time (32 vs. 115 s; *p* < 0.001), while more leads were extracted per procedure (2 vs. 1; *p* < 0.001); ≥2 leads were removed in 19 of 27 infective versus 6 of 27 non-infective procedures (70% vs. 22%). Graded according to the 2018 EHRA consensus definitions [15], complete procedural success—radiological and clinical—was achieved in 50 of 54 procedures (92.6%) and clinical success in 51 (94.4%): one procedure with a retained ventricular lead fragment achieved clinical but not radiological success, and extraction failed in three (5.6%); all four incomplete extractions had non-infective indications. An intra-procedural complication was recorded in three procedures (5.6%), all with non-infective indications; none was permanently disabling.

### 3.2. The Infective Indication Predicts Extraction Extent but Not Technical Complexity

**Figure 2:**
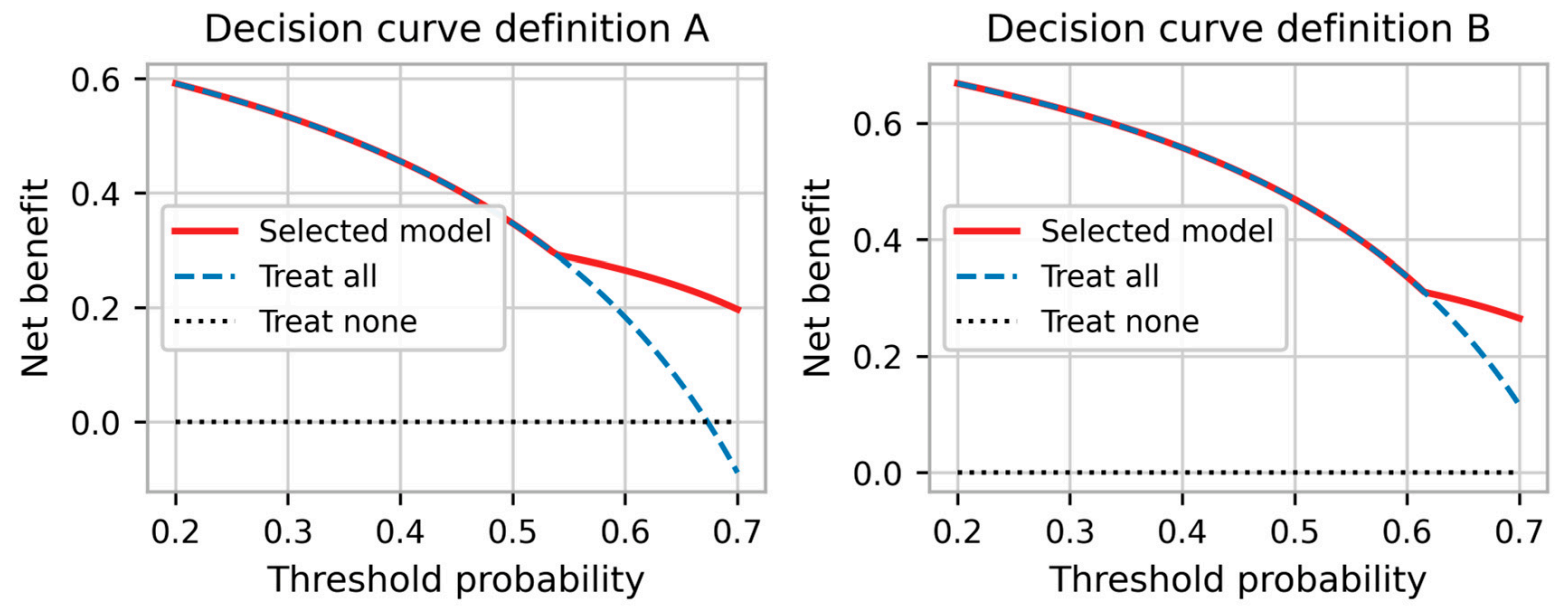
Decision-curve analysis for the extraction-extent endpoints (composite definitions A and B; sensitivity analysis). Net benefit of the BIC-selected model (infective indication) is plotted against treat-all and treat-none strategies across threshold probabilities 0.20–0.70. The model offers modest positive net benefit only at thresholds ≥ 0.55.

Table 1 decomposes the procedural endpoints (panel (a)). Under composite definition A, 85.2% of infective versus 55.6% of non-infective procedures were classified as complex (OR 4.60, *p* = 0.035), and 18 procedures—16 of them infective—met the definition solely through the ≥2-leads criterion (10 and 8, respectively, under composite definition B). Because complete system removal is mandated in infection, this criterion incorporates the indication into the outcome, and the composite definitions are therefore reported as extraction-extent endpoints. On the technical endpoints the association disappeared: prolonged procedure or fluoroscopy time was non-significantly less frequent in infective procedures (25.9% vs. 48.1%; OR 0.38, *p* = 0.16) and advanced-tool use did not differ (59.3% vs. 51.9%; OR 1.35, *p* = 0.79). In prespecified two-predictor models (Table 1, panel (b)), the infective indication predicted neither technical endpoint, whereas cumulative lead dwell time predicted advanced-tool use (OR 1.27 per year, 95% CI 1.06–1.52, *p* = 0.011; apparent AUC 0.75, optimism-corrected 0.72; calibration slope 0.94). BIC-based selection from ten candidates—the routine panel, the indication, lead age, cumulative dwell time and the number of extracted leads—retained no variable for technical definition A and only cumulative dwell time for technical definition B (OR 1.27, 95% CI 1.05–1.55; optimism-corrected AUC 0.74). Excluding the second procedure of the patient extracted twice altered odds ratios by ≤0.13 and changed no conclusion. For the extraction-extent endpoints the corresponding logistic models are reported as sensitivity analyses (Table 1, panel (c)): only the infective indication was retained (composite definition A OR 4.07, 95% CI 1.08–15.33, *p* = 0.038; composite definition B OR 4.17, 95% CI 0.98–17.73, *p* = 0.053; apparent and optimism-corrected AUC 0.66; Hosmer–Lemeshow *p* = 0.74 and 0.85), and decision-curve analysis of these extent models showed net benefit over treat-all only at threshold probabilities ≥0.55 (Figure 2).

### 3.3. Exploratory Bleeding-Proxy Model

In the absence of a directly captured bleeding endpoint, the exploratory DIFFICULT proxy model (nine candidate predictors; 38 complete cases; eight non-events) cannot support inference about predictive performance and is reported only in the Supplementary Materials (Sections S5 and S8), where it is labelled hypothesis-generating. No bleeding-related conclusion is drawn from it.

### 3.4. Peri-Procedural MMP-9 Dynamics Do Not Reflect Baseline Haemostasis or Inflammation

MMP-9 did not change significantly between pre and post measurements in the overall cohort (median Δ% +18.9%; Wilcoxon W = 383, *p* = 0.13) or within indication strata (non-infective Δ% −0.02%, *p* = 0.57; infective Δ% +23.0%, *p* = 0.22). Spearman correlations of MMP-9 percentage change with baseline PT, INR, APTT, platelets, haemoglobin and CRP were all small (|ρ| ≤ 0.25) and none survived BH adjustment (lowest q = 0.37). A multivariable HC3-robust linear regression on CRP, platelets, haemoglobin, the infective indication, log procedure time and number of leads extracted retained no predictor at *p* < 0.05 (all *p* ≥ 0.30). Residuals were non-normal (Shapiro–Wilk *p* = 2 × 10^−8^) with a high maximum standardised residual (4.6) and a high maximum Cook’s distance (0.72), reflecting one influential outlier and limiting inferential strength. In sensitivity analyses addressing this, winsorisation of continuous variables at the 1st/99th percentiles and leave-one-out refits changed no coefficient’s inferential status.

### 3.5. Descriptive Comparison by Indication: The Panel and MMP-9 Do Not Differ Between Infective and Non-Infective Extraction

**Figure 3:**
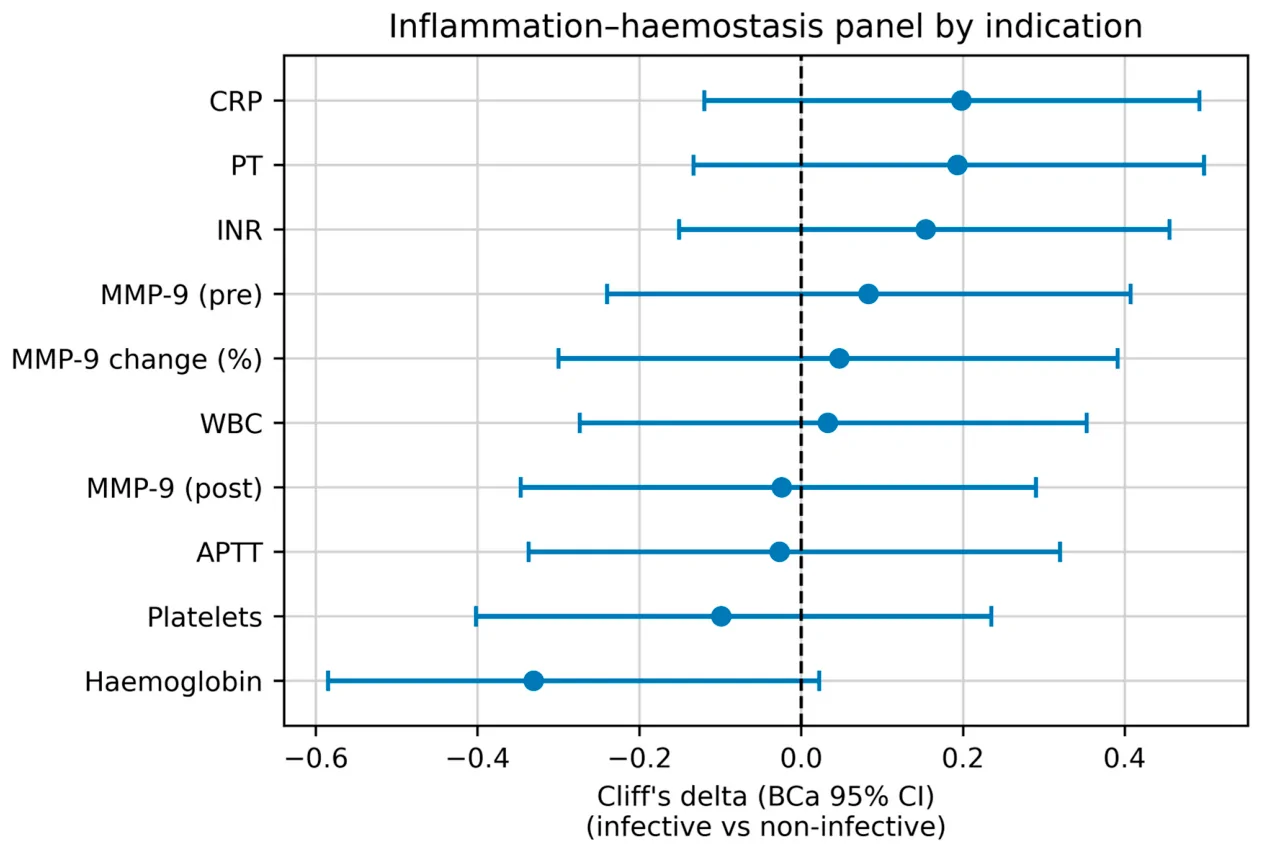
Forest plot of rank-based effect sizes (Cliff’s delta with BCa bootstrap 95% CI) for the inflammation–haemostasis panel and MMP-9 between infective and non-infective indications; Hedges’ g with BCa CIs is reported in Table 2. Only haemoglobin reaches nominal significance (Mann–Whitney *p* = 0.038); its interval for Cliff’s delta nonetheless marginally includes zero, as bootstrap intervals need not coincide with the exact rank-sum test at the margin. No variable survives Bonferroni or Benjamini–Hochberg adjustment.

Reported here as a descriptive basic-science observation rather than as a diagnostic test, group comparisons of the panel are summarised in Table 2 and Figure 3. The infective group had nominally lower haemoglobin (13.0 vs. 14.0 g/dL; *p* = 0.038; Hedges’ g −0.63, BCa 95% CI −1.20 to −0.07; Cliff’s delta −0.33, 95% CI −0.59 to 0.02; HL difference −1.0 g/dL, 95% CI −2.0 to −0.1). The BCa interval for Cliff’s delta marginally includes zero despite the nominally significant rank-sum test; bootstrap intervals are not exact test inversions, and this borderline disagreement underscores the fragility of the haemoglobin signal. No other panel variable showed a nominal between-group difference (all *p* ≥ 0.22). After Bonferroni correction (k = 10) or BH adjustment, no variable retained statistical significance (lowest BH q = 0.38). For CRP the mean-based effect size was positive (g 0.59; BCa 95% CI 0.19 to 0.93) whereas rank-based measures showed no difference (Cliff’s delta 0.20, 95% CI −0.12 to 0.49; Mann–Whitney *p* = 0.22; HL difference 2.25 mg/L, 95% CI −1.0 to 10.35): a small number of high CRP values in the infective group inflates the mean-based difference, and rank-based measures are therefore emphasised. Pre-procedural MMP-9 (g 0.12; *p* = 0.62), post-procedural MMP-9 (g −0.09; *p* = 0.89) and MMP-9 percentage change (g −0.35; *p* = 0.79) did not differ between groups.

### 3.6. A Principal-Component Composite Adds No Information over CRP Alone

**Figure 4:**
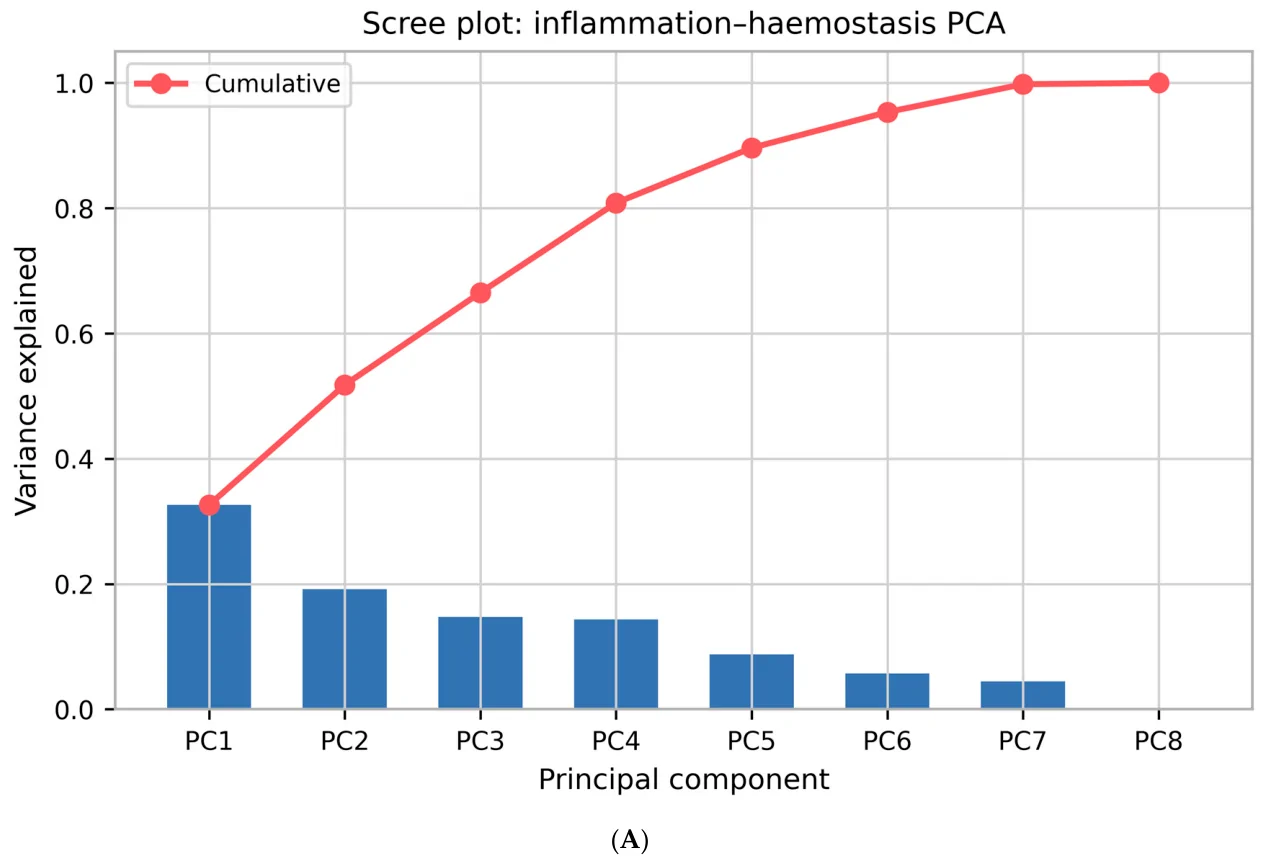

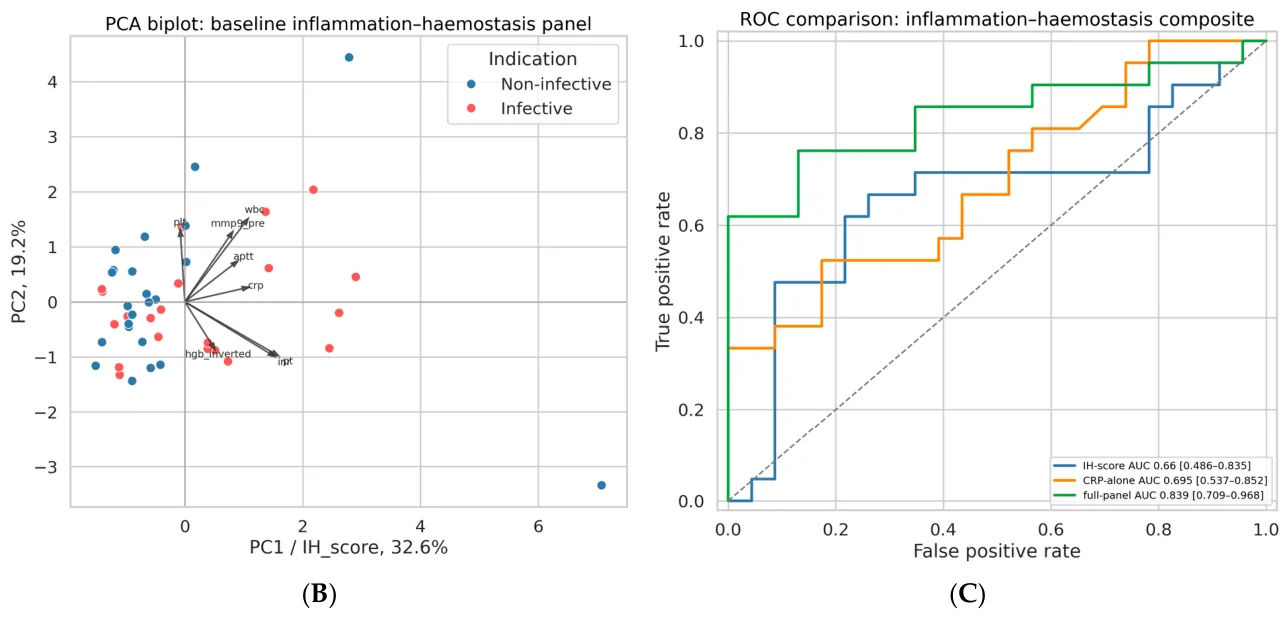
Principal-component analysis. (**A**) Scree plot. (**B**) PC1–PC2 biplot. (**C**) Receiver-operating-characteristic curves for the infective indication: IH score (PC1) AUC 0.66, CRP alone AUC 0.69, full multivariable panel AUC 0.84. DeLong test: IH vs. CRP *p* = 0.67; IH vs. full panel *p* = 0.10.

PCA on the eight z-scored panel variables (haemoglobin inverted) yielded PC1, accounting for 32.6% of variance, and PC2 for 19.2%, with PT and INR dominating PC1 loadings (0.53 and 0.51) followed by CRP, white-cell count, APTT, MMP-9 and the inverted haemoglobin (Table 3; Figure 4A,B). The PC1-derived IH score did not discriminate the infective indication (AUC 0.66, 95% CI 0.49–0.83) and was statistically indistinguishable from CRP alone (AUC 0.69, 95% CI 0.54–0.85; DeLong difference −0.034, *p* = 0.67) (Figure 4C). The full multivariable panel reached AUC 0.84 (95% CI 0.71–0.97); the DeLong comparison between the IH score and the full panel was not significant (Δ = −0.18, *p* = 0.10). After 1000-resample optimism correction, the full-panel AUC fell to 0.71 (calibration slope 0.42) and is reported as descriptive only; in a sensitivity PCA excluding INR (seven variables), PC1 explained 30.4% of variance and conclusions were unchanged (PC1 AUC 0.64 vs. CRP alone 0.69; DeLong *p* = 0.44). The IH-score logistic regression for the infective indication produced OR 1.26 per 1-unit increase (95% CI 0.84–1.91; *p* = 0.27) and OR 1.37 per IQR increase (95% CI 0.78–2.41; *p* = 0.27).

## 4. Discussion

In this prospective single-centre cohort of 53 patients undergoing 54 transvenous lead extraction procedures, no component of the routine pre-procedural inflammation–haemostasis panel—PT, INR, APTT, platelets, haemoglobin, white-cell count, CRP, with MMP-9 as a candidate bridge marker—predicted technical intra-procedural complexity; the association of the infective indication with complexity reflected extraction extent (guideline-mandated complete system removal) rather than technical difficulty. Only cumulative lead dwell time predicted the need for advanced extraction tools, and peri-procedural MMP-9 change was modest and unrelated to baseline haemostasis or inflammation. As a descriptive observation, the inflammatory–haemostatic milieu, including MMP-9, did not differ between the infective and non-infective indications after correction for multiple testing. These exploratory findings carry three implications.

### 4.1. Routine Laboratory Tests Are Insufficient Pre-Procedural Risk Markers

Our findings align with two strands of contemporary evidence. Recent multicentre and registry analyses have repeatedly shown that routine inflammatory markers have limited sensitivity for the diagnosis of cardiac implantable electronic device (CIED) pocket infection [16,17]; A prospective multicentre case–control evaluation reported a sensitivity of 60% for procalcitonin and similarly modest performance for CRP in isolated pocket infection [17]. In parallel, the ELECTRa antithrombotic-therapy sub-analysis demonstrated that minimised peri-procedural antithrombotic management is safe and that the major-complication rate is driven by indication and lead burden rather than by baseline coagulation parameters [14]. The present data extend these observations to the pre-procedural setting: an unselected inflammation–haemostasis panel measured the day before extraction does not predict technical intra-procedural complexity, and the infective indication itself predicts extraction extent rather than technical difficulty. Consistent with this, and reported here only as a descriptive observation, the same panel did not differ between the infective and non-infective indications—in agreement with earlier series on the limited haematological signature of CIED infection [16,17].

Our descriptive between-indication comparison is consistent with the recent retrospective analysis of 156 TLE patients by Lacharite-Roberge and colleagues, in which procalcitonin discriminated systemic from isolated pocket infection (*p* = 0.009) but did not separate isolated pocket infection from controls, and in which CRP and white-cell count had only modest differential performance [18]. Our prospective design adds formal Bonferroni and Benjamini–Hochberg correction, bootstrap optimism correction and decision-curve analysis that were not available in that retrospective series. We emphasise that the clinically relevant question here is not diagnostic—the indication is known before extraction—but prognostic: identifying which patients face a complex, higher-bleeding-risk procedure. The convergence of two independent cohorts indicating that the routine inflammatory panel carries little indication-specific signal supports the methodological position of the EHRA–ISCVID–ESCMID consensus [3] that fibrosis-biomarker and haemostasis-activation panels, rather than routine baseline screening tests, are required for clinical decision-making in this setting. Kosior and colleagues reported a significant rise in cardiac troponin T after TLE in a prospective cohort and identified CRP as an independent predictor of major complications in a prior 504-patient series [19]. The Tajstra and colleagues SILCARD-registry data documented that the all-cause mortality of patients with TLE-related complications was 23.9% versus 6.5% in those without complications [20], reinforcing that the catastrophic-bleeding subgroup drives the mortality signal and is precisely the subgroup our routine panel does not capture. A directed combined panel—D-dimer, fibrinogen, von Willebrand factor, troponin T and a fibrosis-biomarker tier—is the logical next step.

### 4.2. Anaemia Is the Only Nominal Signal, Consistent with SAFeTY-TLE

The single nominal between-group difference observed in our cohort was a 1.0 g/dL lower median haemoglobin concentration in patients with the infective indication. Anaemia is established as a long-term mortality predictor after TLE [21,22] and is one of the five canonical components of the SAFeTY-TLE score developed by the Polish group [4]. The observation that this nominal signal does not survive multiple-testing correction, and that haemoglobin does not enter the stepwise complexity model once the infective indication is allowed in, is biologically consistent: anaemia tracks systemic infection rather than providing independent predictive information beyond the indication itself. The data therefore reinforce the design of SAFeTY-TLE, in which anaemia is one of several composite components, rather than supporting a standalone haemoglobin threshold for risk stratification. Age and comorbidity interact with this picture: in a recent elderly TLE cohort (median age 76 years), anaemia and atrial fibrillation—not the extraction indication itself—independently predicted long-term mortality [23]. Read together with our data, anaemia marks the older, comorbid and often infected phenotype—with greater arrhythmia burden and lower ejection fraction—rather than acting as an independent laboratory predictor of procedural difficulty, and supports a rapid and effective extraction strategy in aging patients rather than laboratory-based triage [23].

### 4.3. A Composite IH Score Does Not Outperform CRP Alone

PCA-derived composite scores have been proposed for risk stratification in inflammatory states, but in this cohort the IH score (PC1, dominated by PT, INR, CRP and white-cell count) added no discriminatory information over CRP alone (DeLong *p* = 0.67). The point estimate for the full multivariable panel reached AUC 0.84 (optimism-corrected 0.71), but the comparison with the IH score did not reach formal significance (DeLong *p* = 0.10). Three conclusions follow: in this sample size, panel-derived composites do not advance pre-procedural risk assessment for the infective indication; CRP, despite its known limitations, remains the most parsimonious single laboratory marker that approaches a useful AUC; and a substantially augmented model—likely incorporating fibrosis biomarkers such as galectin-3, sST2 and TGF-β1—appears required to reach clinically actionable discrimination.

### 4.4. MMP-9 Dynamics Are Not Paralleled by Haemostasis Activation

**Figure 5:**
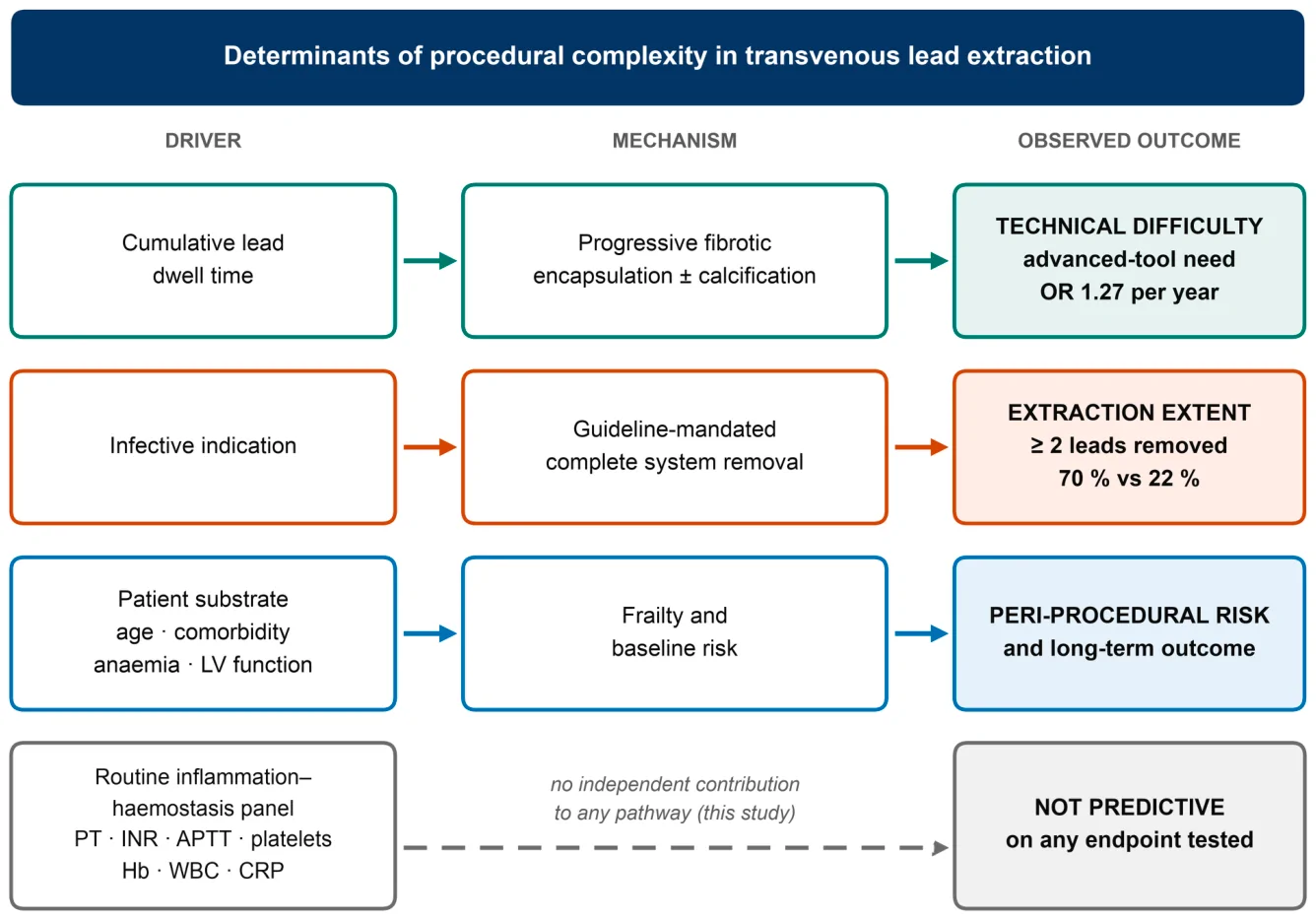
Determinants of procedural complexity in transvenous lead extraction suggested by the present data. Cumulative lead dwell time drives fibrotic encapsulation and technical difficulty (advanced-tool need); the infective indication mandates complete system removal and therefore extraction extent; patient substrate shapes peri-procedural risk; the routine inflammation–haemostasis panel contributed independently to none of these pathways.

MMP-9 rises sharply two to six hours after the start of cardiopulmonary bypass and reflects neutrophil-mediated extracellular matrix remodelling under systemic inflammatory stress [24,25]. In TLE, however, mechanical stress is more localised and shorter, and the present cohort showed only a non-significant median rise of approximately 19%, with no correlation between MMP-9 percentage change and baseline PT, INR, APTT, platelets, haemoglobin or CRP after false-discovery-rate (FDR) adjustment. This contrasts with systemic surgical-bypass biology and is consistent with the more contained mechanical insult of percutaneous lead extraction. As a bridge biomarker between inflammation and haemostasis, MMP-9 therefore does not appear to serve a quantitative role in this procedure, and the haemostasis side of the inflammation–haemostasis axis cannot be inferred from MMP-9 dynamics alone. By contrast, cumulative lead dwell time—the principal driver of fibrotic lead encapsulation [26,27]—was the only variable retained for advanced-tool use by BIC selection. This is mechanistically expected: beyond the first year after implantation, traction alone rarely suffices, and no single technique is sufficient for long-dwelling leads [28]. Figure 5 summarises the determinants of procedural complexity suggested by these data: dwell-time-driven fibrosis governs technical difficulty; the infective indication governs extraction extent; patient substrate governs peri-procedural risk; and routine laboratory screening contributes independently to none of these pathways.

### 4.5. Implications for Risk Stratification and the Design of Future Studies

The results have three practical implications. The absence of haemostasis-activation markers (D-dimer, prothrombin fragment 1 + 2, fibrinogen, von Willebrand factor) is the principal mechanistic limitation of the present panel: every parameter we measured is a baseline screening test, not an activation marker. D-dimer in particular has well-described peri-procedural dynamics in cardiac and thoracic surgery and tracks fibrinolytic activation [29,30], while fibrinogen drop and platelet activation accompany clinically relevant bleeding. A directed haemostasis-activation panel measured before, immediately after and at 24 h after TLE is therefore a logical next step. In parallel, intra-procedural bleeding endpoints must be explicitly captured (volume of blood loss, transfusion requirement, pericardial drainage) rather than approximated by fluoroscopy time or tool use; the bleeding-proxy model—now reported only in the Supplementary Materials as hypothesis-generating—is calibration-limited and AUC-wide, reflecting the absence of a directly captured haemorrhagic endpoint, and no bleeding-related inference is drawn from it. Given the dominance of vascular complications in TLE mortality and the small but real proportion of patients receiving combined antithrombotic therapy, a prospective multicentre study with paired baseline and activation markers, explicit bleeding capture and stratification by antithrombotic regimen is the appropriate design—ideally embedded within an established registry such as the ELECTRa platform [1,7,14]. Disposition decisions illustrate the same principle: same-day discharge after uncomplicated TLE has proved feasible and safe in selected—predominantly non-infective—patients [31], resting on procedural rather than laboratory criteria.

### 4.6. Limitations

This is an exploratory single-centre prospective cohort of 53 patients (54 procedures) with MMP-9 available in 45–50 of the 54 procedures depending on the sampling point; one patient contributed two procedures, and a sensitivity analysis excluding the second changed no conclusion. Pre-procedural haemostasis was characterised by routine laboratory screening tests, not by activation markers (D-dimer, fibrinogen, F1 + 2, von Willebrand factor), and intra-procedural bleeding was not directly measured but approximated through fluoroscopy time, procedure time, advanced-tool use and the number of leads extracted. The multivariable models are exploratory; the optimism-corrected AUCs of the technical models (0.56 for definition A and 0.72–0.74 for definition B) and of the extraction-extent models (0.66) fall short of clinical actionability. Several non-normal distributions and one or two influential outliers reduce inferential confidence in the linear MMP-9 model. Success grading relies on the registry’s radiological and clinical success flags; no complication was permanently disabling, but the nature of the three intra-procedural complications was not otherwise recorded. Infection subtypes are reported descriptively, and the infective subgroup is too small for subtype-stratified analyses. External validation in a multicentre cohort, ideally with prespecified activation markers and explicit bleeding endpoints, is essential before any clinical adoption. The composite complexity definitions incorporate the number of extracted leads and thereby partly encode the indication; they are therefore analysed only as extraction-extent sensitivity endpoints, and the technical endpoints—though free of incorporation bias—remain surrogates for operative difficulty. Consistent with the power analysis in Section 2.5, the absence of statistical significance here reflects absence of evidence at this sample size rather than evidence of absence.

## 5. Conclusions

In this exploratory single-centre cohort, no routine pre-procedural inflammation–haemostasis variable predicted technical intra-procedural complexity in transvenous lead extraction, and peri-procedural MMP-9 dynamics were modest and unrelated to any baseline haemostasis marker. The apparent predictive value of the infective indication for procedural complexity reflected guideline-mandated complete system removal—extraction extent—rather than technical difficulty; only cumulative lead dwell time predicted the need for advanced extraction tools. Descriptively, the inflammatory–haemostatic milieu, including MMP-9, did not differ between the infective and non-infective indications; the single nominal signal—lower haemoglobin in the infective group—did not survive multiple-testing correction and is consistent with the SAFeTY-TLE composite rather than providing independent information. A principal-component composite added nothing to CRP alone, and the full multivariable panel’s apparent discrimination shrank substantially after optimism correction. These findings reinforce the inability of routine laboratory tests to substitute for dedicated risk scoring—anchored in anatomical and dwell-time-related factors—or for a directed haemostasis-activation and fibrosis-biomarker panel, and motivate a prospective study incorporating explicit intra-procedural bleeding-event capture.

## Figures and Tables

**Table 1:** Procedural endpoints by indication (a), models for technical complexity (b) and extraction-extent sensitivity analyses (c).

*(a) Endpoint decomposition.*							
**Endpoint**	**n**	**Complex—infective**	**Complex—non-infective**	**OR**	***p***		
Technical A (time/fluoroscopy)	54	7/27 (25.9%)	13/27 (48.1%)	0.38	0.158		
Technical B (advanced tools)	54	16/27 (59.3%)	14/27 (51.9%)	1.35	0.785		
Extent A (composite definition A)	54	23/27 (85.2%)	15/27 (55.6%)	4.60	0.035		
Extent B (composite definition B)	54	24/27 (88.9%)	16/27 (59.3%)	5.50	0.028		
*(b) Models for technical complexity.*							
**Endpoint**	**Model**	**Term**	**OR (95% CI)**	***p***	**AUC apparent**	**AUC corrected**	**Calibration slope**
Technical A	Prespecified	Infective indication	0.38 (0.12–1.18)	0.095	0.61	0.56	0.76
Technical A	Prespecified	Dwell time (per year)	1.00 (0.87–1.14)	0.939			
Technical A	BIC selection	None retained	—	—	—	—	—
Technical B	Prespecified	Infective indication	1.65 (0.51–5.37)	0.407	0.75	0.72	0.94
Technical B	Prespecified	Dwell time (per year)	1.27 (1.06–1.52)	0.011			
Technical B	BIC selection	Dwell time (per year)	1.27 (1.05–1.55)	0.015	0.74	0.74	—
*(c) Extraction-extent sensitivity analyses (composite definitions A and B).*							
**Endpoint**	**Term**	**OR (95% CI)**	***p***	**AUC apparent (95% CI)**	**AUC corrected (95% CI)**	**Hosmer–Lemeshow *p***	
Extent A	Infective indication	4.07 (1.08–15.33)	0.038	0.66 (0.52–0.80)	0.66 (0.51–0.78)	0.74	
Extent B	Infective indication	4.17 (0.98–17.73)	0.053	0.66 (0.52–0.81)	0.66 (0.50–0.79)	0.85	

Technical A: fluoroscopy time > 75th percentile (131 s) OR procedure time > 75th percentile (100 min). Technical B: Byrd OR Evolution sheath. Extent A/B: composite definitions identical to technical A/B but additionally counting ≥2 extracted leads as complex; under extent A, 18 procedures (16 infective) qualified solely via the ≥2-leads criterion, under extent B, 10 (8 infective). Panel (a): Fisher exact tests. Panel (b): prespecified models *n* = 54; BIC selection from ten candidates, *n* = 49 complete cases; optimism correction and calibration slope from 1000 bootstrap resamples; no separation occurred, so maximum-likelihood estimates are reported; excluding the second procedure of the patient extracted twice changed odds ratios by ≤0.13. Panel (c): extraction-extent models (*n* = 49).

**Table 2:** Inflammation–haemostasis panel by indication.

Variable	n Non-Inf/Inf	Non-Infective Median (IQR)	Infective Median (IQR)	*p*	g (BCa 95% CI)	HL Diff (95% CI)	BH q
PT, s	27/27	12.1 (11.4–12.6)	12.5 (11.8–14.25)	0.225	−0.11 (−0.59, 0.48)	0.41 (−0.29, 1.40)	0.75
INR	27/27	1.04 (0.99–1.12)	1.08 (1.01–1.28)	0.336	−0.12 (−0.60, 0.56)	0.03 (−0.03, 0.12)	0.84
APTT, s	27/23	29.9 (28.9–34.75)	30.8 (28.15–34.85)	0.876	−0.32 (−0.73, 0.24)	−0.20 (−3.3, 2.4)	0.89
Hgb, g/dL	27/27	14.0 (13.2–14.6)	13.0 (11.59–14.45)	0.038 *	−0.63 (−1.20, −0.07)	−1.00 (−2.0, −0.1)	0.38
PLT, ×10^9^/L	27/27	243 (189–269)	203 (173–276.5)	0.539	−0.08 (−0.65, 0.45)	−11 (−58, 30)	0.89
WBC, ×10^9^/L	27/27	7.93 (6.62–9.40)	8.13 (6.49–9.55)	0.842	0.02 (−0.53, 0.55)	0.10 (−1.05, 1.31)	0.89
CRP, mg/L	26/27	6.40 (2.58–11.93)	10.80 (2.50–25.15)	0.220	0.59 (0.19, 0.93)	2.25 (−1.00, 10.35)	0.75
MMP-9 pre, ng/mL	24/25	9.21 (6.64–11.04)	9.66 (6.72–12.61)	0.624	0.12 (−0.49, 0.70)	0.61 (−1.98, 3.23)	0.89
MMP-9 post, ng/mL	25/25	10.34 (7.85–15.69)	9.63 (7.49–12.67)	0.892	−0.09 (−0.66, 0.47)	−0.41 (−3.07, 2.10)	0.89
MMP-9 Δ%	22/23	−0.02 (−11.4, 57.4)	23.0 (−10.2, 52.2)	0.794	−0.35 (−0.77, 0.25)	+6.2 (−38.0, 36.0)	0.89

Median (IQR) for continuous variables; Mann–Whitney U test; standardised mean difference (Hedges’ g) with BCa bootstrap 95% CI (2000 resamples); Hodges–Lehmann (HL) difference (infective − non-infective) with 95% CI; multiple-testing adjustment by Bonferroni (k = 10) and Benjamini–Hochberg (BH). * nominal *p* < 0.05. No variable significant after Bonferroni or BH.

**Table 3:** Principal-component analysis: variance explained and PC1 loadings.

Variable	PC1 Loading	PC2 Loading
PT	0.531	−0.327
INR	0.511	−0.333
CRP	0.365	0.089
WBC	0.359	0.506
APTT	0.301	0.249
MMP-9 pre	0.272	0.427
Hgb inverted	0.171	−0.290
PLT	−0.026	0.437
Variance explained	32.6%	19.2%

PCA on z-scored variables (haemoglobin inverted). Cumulative variance for the first two components 51.8%.

## Data Availability

Anonymised patient-level and lead-level datasets supporting the conclusions of this article will be made available by the corresponding author upon reasonable request, subject to approval by the Bioethics Committee of the Medical University of Warsaw.

## Supplementary Materials

The following supporting information can be downloaded at https://www.mdpi.com/article/10.3390/jcm15176923/s1, S1, Atrial-fibrillation-stratified haemostasis panel as a proxy for chronic anticoagulation; S2, Detailed principal-component analysis of the inflammation–haemostasis panel; S3, AUC of the IH score vs CRP alone vs the full multivariable panel for the infective indication, with DeLong comparison; S4, Full decision-curve net-benefit table across threshold probabilities 0.20–0.70 for the extraction-extent endpoints (composite definitions A and B); S5, Composite DIFFICULT endpoint: component breakdown and sensitivity to alternative cut-points; S6, Inter-haemostasis-marker Spearman correlation matrix; S7, MMP-9 percentage change: multivariable HC3-robust linear regression and residual diagnostics; S8, DIFFICULT-proxy bleeding model: full logistic detail, calibration deciles, ROC and Hosmer–Lemeshow test.

## Abbreviations

APTT	activated partial thromboplastin time
AUC	area under the receiver-operating-characteristic curve
BCa	bias-corrected and accelerated (bootstrap confidence interval)
BH	Benjamini–Hochberg
BIC	Bayesian information criterion
CIED	cardiac implantable electronic device
CRP	C-reactive protein
EHRA	European Heart Rhythm Association
ELECTRa	European Lead Extraction ConTRolled registry
EROS	ELECTRa Registry Outcome Score
FDR	false discovery rate
HL	Hodges–Lehmann
IH	inflammation–haemostasis
INR	international normalised ratio
IQR	interquartile range
LED	Lead Extraction Difficulty
LDIE	lead-dependent infective endocarditis
LVEF	left-ventricular ejection fraction
MMP-9	matrix metalloproteinase-9
NT-proBNP	N-terminal pro-B-type natriuretic peptide
PADIT	Prior procedures, Age, Depressed renal function, Immunocompromised, Type of procedure
PCA	principal-component analysis
PT	prothrombin time
SAFeTY-TLE	Sum of dwell times, Anaemia, Female sex, Treatment history, Younger age
TLE	transvenous lead extraction
