# Supplementary material for: Routine Inflammation–Haemostasis Panel in Transvenous Lead Extraction: An Exploratory Prospective Single-Centre Cohort Study
Source: J Clin Med. 2026 Sep 7;15(17):6923. doi: 10.3390/jcm15176923 (PMC13566128; doi:10.3390/jcm15176923)
Supplement: Supplementary file 1 [file jcm-15-06923-s001.zip › jcm-4524459-supplementary.pdf]

Supplementary Materials

Routine inflammation–haemostasis panel in transvenous lead extraction: an exploratory prospective single-centre cohort study.

Authors: Jakub Rokicki[1,2], Sylwia Wolff[3], Łukasz Januszkiewicz[2], Marcin Michalak[2] Katarzyna Czarzasta[4], Andrzej Cacko[1], Grzegorz Opolski[2], Marcin Grabowski[2]

Note added at revision: one enrolled participant withdrew consent with no data collected (main-text Figure 1); all supplementary denominators refer to the 54-procedure analysis set (53 patients). Analyses are reported as originally specified, and the DIFFICULT cut-points retain their originally derived values (fluoroscopy 75th/90th percentiles, 135.0 s and 225.0 s).

S1. Atrial-fibrillation-stratified haemostasis panel as a proxy for chronic anticoagulation

Methods. Explicit DOAC / VKA exposure was not captured in the cleaned cohort dataset; atrial fibrillation (AF), present in 33 of 54 procedures (61 %), was used as a proxy for the probability of chronic anticoagulation. Continuous variables of the inflammation–haemostasis panel were compared between AF (n = 33) and no-AF (n = 21) groups by the Mann–Whitney U test. No multiplicity correction was applied (planned sensitivity analysis).

Results. Of eight panel variables, three reached nominal $p < 0.05$ — PT (12.50 vs 11.80 s, p = 0.021), INR (1.09 vs 1.01, p = 0.011) and APTT (32.70 vs 29.15 s, p = 0.026) — all in the direction expected of vitamin-K-antagonist or DOAC exposure. Platelets, haemoglobin, white-cell count, CRP and pre-procedural MMP-9 did not differ between AF and no-AF ($p \geq 0.34$).

Variable	AF (n=33)	No AF (n=21)	Mann–Whitney p
PT, s	12.50 (11.90–14.10)	11.80 (11.20–12.50)	0.021
INR	1.09 (1.04–1.27)	1.01 (0.98–1.08)	0.011
APTT, s	32.70 (29.43–36.05)	29.15 (27.98–30.82)	0.026
Platelets, ×10⁹/L	227 (183–282)	203 (175–271)	0.683
Haemoglobin, g/dL	13.50 (12.05–14.50)	14.00 (12.60–14.70)	0.632
CRP, mg/L	7.75 (2.65–16.40)	6.40 (2.50–12.20)	0.643
WBC, ×10⁹/L	7.93 (6.20–9.58)	8.42 (6.70–9.30)	0.342
MMP-9 pre, ng/mL	9.44 (7.06–11.41)	8.52 (6.40–12.47)	0.870

Interpretation. *AF-driven anticoagulation prolongs PT/INR/APTT as expected but does not eclipse the main-analysis finding that the haemostasis panel does not discriminate the infective*

from the non-infective TLE indication. Direct DOAC / VKA exposure should be captured prospectively in future cohorts and incorporated as a stratification variable in any future multivariable model.

S2. Detailed principal-component analysis of the inflammation–haemostasis panel

Methods. Principal-component analysis (PCA) was applied to the eight z-scored variables of the inflammation–haemostasis panel (PT, INR, APTT, platelets, haemoglobin [inverted], white-cell count, CRP, pre-procedural MMP-9) using a complete-case subset (n = 44). PC scores were standardised. Variance explained, cumulative variance and component loadings are reported below; the PC1 ('IH score') is the composite used in the main-text Section 4.3 model comparison.

Variance explained per component:

Component	Variance explained	Cumulative variance
PC1	32.6 %	32.6 %
PC2	19.2 %	51.8 %
PC3	14.7 %	66.5 %
PC4	14.3 %	80.8 %
PC5	8.8 %	89.6 %
PC6	5.7 %	95.3 %
PC7	4.5 %	99.8 %
PC8	0.2 %	100.0 %

Variable loadings on PC1 and PC2 (highest absolute loading per row in bold):

Variable	PC1 loading	PC2 loading
PT	+0.531	−0.327
INR	+0.511	−0.333
CRP	+0.365	+0.089
WBC	+0.359	+0.506
APTT	+0.301	+0.249
MMP-9 pre	+0.272	+0.427
Haemoglobin (inverted)	+0.171	−0.290
Platelets	−0.026	+0.437

Interpretation. *PC1 is dominated by the coagulation-cascade axis (PT, INR) with secondary contributions from CRP and white-cell count; PC2 carries an orthogonal 'cellular-inflammation / platelet-WBC / MMP-9' signature. The minimal PC1 weight of platelets and inverted haemoglobin indicates that anaemia and thrombocytopenia status of the cohort are not captured by the first principal component — which is why a PC1-only IH score does not outperform CRP alone (S3).*

S3. AUC of the IH score vs CRP alone vs the full multivariable panel for the infective indication, with DeLong comparison

Methods. Three nested-but-different logistic-regression models for the infective indication were fitted on the complete-case subset (n = 44, events = 21): (i) IH-score (PC1) alone; (ii) CRP alone; (iii) the full multivariable panel of eight inflammation–haemostasis variables. AUCs are reported with their parametric 95 % CI (Hanley–McNeil); pairwise comparison of the IH-score model with the two alternatives used the DeLong test.

Model	N / events	AUC (95% CI)	SE	DeLong vs IH (p)
IH score (PC1)	44 / 21	0.660 (0.486–0.835)	0.089	—
CRP alone	44 / 21	0.695 (0.537–0.852)	0.080	0.667
Full panel (all 8 variables)	44 / 21	0.839 (0.709–0.968)	0.066	0.105

Interpretation. *The PC1-based IH composite (AUC 0.660) provides no statistically significant advantage over CRP alone (AUC 0.695; DeLong p = 0.667). The full multivariable panel reaches AUC 0.839 but its margin over the IH score is also not statistically significant in this sample size (DeLong p = 0.105). The simplest parsimonious laboratory marker that approaches the panel performance is therefore CRP, with the multivariable model offering a numerical (but not formally significant) gain. Both fall short of the discrimination that a fibrosis-biomarker panel would be expected to provide.*

S4. Full decision-curve net-benefit table across threshold probabilities 0.20–0.70 for the extraction-extent endpoints (composite definitions A and B)

Methods. Decision-curve analysis was performed on the BIC-stepwise-selected logistic model for each of the two extraction-extent composite definitions (A: fluoroscopy > 75th percentile OR procedure time > 75th percentile OR ≥ 2 leads extracted; B: requirement for Byrd OR Evolution OR ≥ 2 leads extracted). For each threshold probability between 0.20 and 0.70 we report the net benefit of the model versus the treat-all and treat-none strategies. Below, a sparse subset is shown; the full curve (101 thresholds × 2 definitions = 202 rows) is available from the corresponding author on request.

Threshold	A: model NB	A: treat-all NB	A: treat-none	B: model NB	B: treat-all NB	B: treat-none
0.20	0.592	0.592	0.000	0.668	0.668	0.000
0.30	0.534	0.534	0.000	0.621	0.621	0.000
0.40	0.456	0.456	0.000	0.558	0.558	0.000
0.50	0.347	0.347	0.000	0.469	0.469	0.000
0.55	0.290	0.282	0.000	0.430	0.418	0.000
0.60	0.265	0.184	0.000	0.337	0.337	0.000
0.65	0.236	0.067	0.000	0.295	0.221	0.000
0.70	0.197	−0.088	0.000	0.265	0.116	0.000

Net-benefit range across the full curve:

Definition	Model NB min	Model NB max	Treat-all NB min	Treat-all NB max
A	0.197 (at 0.70)	0.592 (at 0.20)	−0.088 (at 0.70)	0.592 (at 0.20)
B	0.265 (at 0.70)	0.668 (at 0.20)	0.116 (at 0.70)	0.668 (at 0.20)

Interpretation. *For both definitions the BIC-selected model (in which only the infective indication is retained) is statistically indistinguishable from a treat-all strategy at threshold probabilities ≤ 0.55, and provides only a modest positive net benefit over treat-all at higher thresholds. The benefit is most marked at thresholds 0.60–0.70, i.e. when the clinical cost of an unnecessary 'treat' decision is high — for example, when 'treat' corresponds to referral to a higher-volume centre. In the low-to-moderate threshold range relevant to most procedural-planning decisions, the haemostasis-panel-based model offers no advantage over treating all extractions as potentially complex.*

S5. Composite DIFFICULT endpoint: component breakdown and sensitivity to alternative cut-points

Note added at revision: the DIFFICULT proxy was demoted from the main text at peer review; it is retained here as hypothesis-generating only, with no bleeding-related inference drawn from it (see Methods 2.4 of the revised manuscript).

Methods. The primary DIFFICULT composite (see main-text Section 3.3) was defined as: Byrd dilator OR Evolution rotational sheath OR Liberator locking stylet OR fluoroscopy time > 75th percentile (135.0 s in this cohort) OR ≥ 2 leads extracted. We report the per-component prevalence and three sensitivity definitions tightening the composite.

Component	Events	N	Prevalence
Byrd dilator	30	54	55.6 %
Liberator locking stylet	34	54	63.0 %
Evolution rotational sheath	0	54	0.0 %
Direct traction	24	54	44.4 %
Fluoroscopy > P75 (135.0 s)	13	54	24.1 %
≥ 2 leads extracted	25	54	46.3 %
DIFFICULT (any of above 5, excluding direct traction)	48	54	88.9 %

Sensitivity definitions:

Definition	Composite rule	Events	Prevalence
DIFFICULT_strict	powered sheath (Byrd OR Evolution) AND fluoroscopy > P75	10	18.5 %
DIFFICULT_no_liberator	Byrd OR Evolution OR fluoroscopy > P75 OR ≥ 2 leads (drop Liberator)	43	79.6 %
DIFFICULT_P90	Byrd OR Evolution OR Liberator OR fluoroscopy > P90 (225.0 s) OR ≥ 2 leads	47	87.0 %

Interpretation. *The primary DIFFICULT composite carries an 89 % event rate, which inflates AUC variance and limits the inferential strength of the bleeding-proxy logistic model (Section S8). Liberator use accounts for the majority of the composite (63 %); dropping Liberator from the rule reduces prevalence to 80 % (still high) without changing the qualitative interpretation. The most discriminating sensitivity definition is the strict one (powered sheath AND fluoroscopy > P75; 19 % prevalence), which would be the appropriate target endpoint for a prospective external validation cohort. The Evolution sheath was not deployed in any case in this single-centre Polish cohort and does not contribute to the composite.*

S6. Inter-haemostasis-marker Spearman correlation matrix

Methods. Pairwise Spearman rank correlations were computed on complete-case pairs (n = 44 to 54 per pair) for the eight variables of the inflammation–haemostasis panel. The matrix justifies the use of PCA (Section S2) for dimension reduction prior to logistic modelling.

	**PT**	**INR**	**APTT**	**PLT**	**Hgb**	**WBC**	**CRP**	**MMP-9 pre**
PT	1.000	+0.924	+0.443	−0.210	−0.087	+0.215	+0.314	+0.234
INR	+0.924	1.000	+0.337	−0.146	−0.075	+0.135	+0.258	+0.173
APTT	+0.443	+0.337	1.000	−0.105	−0.233	−0.074	+0.193	+0.064
PLT	−0.210	−0.146	−0.105	1.000	+0.063	+0.271	−0.014	−0.028
Hgb	−0.087	−0.075	−0.233	+0.063	1.000	+0.278	−0.211	+0.109
WBC	+0.215	+0.135	−0.074	+0.271	+0.278	1.000	+0.296	+0.378
CRP	+0.314	+0.258	+0.193	−0.014	−0.211	+0.296	1.000	+0.541
MMP-9 pre	+0.234	+0.173	+0.064	−0.028	+0.109	+0.378	+0.541	1.000

Interpretation. *The strongest correlation is the expected PT–INR collinearity (ρ = 0.924), confirming that the two variables encode the same coagulation-factor activity and should not be entered jointly into a logistic model. CRP and pre-procedural MMP-9 are moderately correlated (ρ = 0.541), consistent with their shared inflammatory-protease biology. The platelet count is essentially uncorrelated with the coagulation panel ($|\rho| \leq 0.27$ for all other variables), supporting its independent role as a haematological marker. The dimensional structure justifies the use of PCA to combine the eight variables into a parsimonious composite.*

S7. MMP-9 percentage change: multivariable HC3-robust linear regression and residual diagnostics

Methods. Multivariable linear regression of peri-procedural MMP-9 percentage change (mmp9_delta_pct) on CRP, platelets, haemoglobin, the infective indication, procedure time and number of leads extracted was fitted with heteroscedasticity-consistent (HC3) robust standard errors (n = 45 complete cases). Residual diagnostics included Shapiro–Wilk normality, Breusch–Pagan heteroskedasticity, maximum absolute standardised residual and maximum Cook's distance.

Term	β	95% CI	p
Intercept	−1.218	−6.305 to +3.868	0.630
CRP	−0.002	−0.021 to +0.017	0.838
Platelets	+0.004	−0.003 to +0.010	0.296
Haemoglobin	+0.033	−0.286 to +0.353	0.833
Infective indication	−0.438	−1.669 to +0.793	0.476
Procedure time, min	+0.006	−0.006 to +0.018	0.324
Number of leads extracted	+0.137	−0.645 to +0.919	0.724

Residual diagnostics:

Diagnostic	Statistic	p
Shapiro–Wilk normality of residuals	W = 0.690	2.4×10^{-8}
Breusch–Pagan heteroskedasticity	χ^2 = 8.49	0.204
Maximum |standardised residual|	4.64	—
Maximum Cook's distance	0.72	—

Interpretation. *No predictor reached $p < 0.05$; the model is not informative for explaining peri-procedural MMP-9 change. Residuals are strongly non-normal (Shapiro–Wilk $p = 2.4 \times 10^{-8}$) with one or two influential observations (max standardised residual 4.64; max Cook's distance 0.72, which exceeds the conventional 4/n = 0.089 threshold). The Breusch–Pagan test does not flag heteroskedasticity (p = 0.20), so HC3 robust SE are conservative; nonetheless, the residual non-normality limits inferential strength. A quantile-median (τ = 0.5) refit was performed in the primary analysis (not shown here) and gave the same direction of effects with similarly null significance.*

S8. DIFFICULT-proxy bleeding model: full logistic detail, calibration deciles, ROC and Hosmer–Lemeshow test

Note added at revision: this model (nine candidate predictors; 38 complete cases; eight non-events) was removed from the main text at peer review because it cannot support inference about predictive performance; it is reported here for transparency as hypothesis-generating only.

Methods. As a proxy for bleeding risk we fitted an all-in logistic regression of the DIFFICULT endpoint (fluoroscopy > median 73 s OR Byrd OR Evolution) on PT, INR, APTT, platelets, haemoglobin, CRP, age, LVEF and log NT-proBNP. Complete-case sample n = 38, events = 30 (78.9 %). Calibration was assessed by deciles of predicted risk and the Hosmer–Lemeshow test; discrimination by AUC with parametric 95 % CI.

Summary metrics:

Metric	Value
Complete-case n	38
Events	30
Event rate	78.9 %
AUC (DeLong 95 % CI)	0.738 (0.536–0.939)
Hosmer–Lemeshow χ^2 (df = 8)	13.39 (p = 0.099)
Reference median fluoroscopy	73.0 s

Logistic-regression coefficients and odds ratios:

Predictor	β	OR (95% CI)	p
Intercept	−6.94	0.001 (3.5×10^{-12} – 2.7×10^{5})	0.484
PT	−0.141	0.87 (0.17–4.40)	0.865
INR	+4.027	56.1 (2.4×10^{-6} – 1.3×10^{9})	0.642
APTT	+0.105	1.11 (0.83–1.49)	0.481
Platelets	+0.005	1.005 (0.989–1.022)	0.531
Haemoglobin	+0.0004	1.000 (0.547–1.829)	0.999
CRP	−0.038	0.962 (0.912–1.016)	0.163
Age	+0.053	1.054 (0.952–1.168)	0.311
LVEF	−0.012	0.988 (0.933–1.046)	0.679
log NT-proBNP	−0.159	0.853 (0.416–1.750)	0.665

Calibration by deciles of predicted risk:

Decile	n	Mean predicted	Observed event rate
1 (lowest)	4	0.378	0.250

Decile	n	Mean predicted	Observed event rate
2	4	0.676	1.000
3	4	0.745	1.000
4	3	0.782	0.333
5	4	0.805	0.500
6	4	0.834	1.000
7	3	0.868	1.000
8	4	0.900	1.000
9	4	0.933	0.750
10 (highest)	4	0.990	1.000

Interpretation. *No individual predictor reached $p < 0.05$ (smallest $p = 0.16$ for CRP, with an inverse direction). The Hosmer–Lemeshow test is borderline ($\chi^2 = 13.39$, df = 8, $p = 0.099$). The calibration deciles show over-prediction in the lowest two deciles (observed 25 % vs predicted 38 %, and observed 33 % vs predicted 78 % in decile 4), with reasonable agreement at higher deciles. The 95 % CI for AUC (0.536–0.939) is wide due to the small sample size and the very high event rate (79 %). Taken together, this proxy model should be interpreted as exploratory and not as a calibrated tool for the prediction of bleeding events. A prospective study with explicit bleeding-event capture (volume of intra-procedural blood loss, transfusion requirement, pericardial drainage) and a panel of haemostasis-activation markers (D-dimer, fibrinogen, prothrombin fragment 1+2, von Willebrand factor) is the appropriate next step.*
